# Facilitators and Barriers for Initiating Conversations About End of Life

**DOI:** 10.1089/pmr.2022.0042

**Published:** 2022-11-21

**Authors:** Mette Aaby Smith, Anne C. Brøchner, Helene K. Nedergaard, Hanne I. Jensen

**Affiliations:** ^1^ Department of Anesthesiology and Intensive Care, Kolding Hospital, University Hospital of Southern Denmark, Kolding, Denmark.; ^2^ Department of Regional Health Research, University of Southern Denmark, Odense, Denmark.

**Keywords:** advance care directives, communication, decision making, end-of-life conversations, wishes for treatment

## Abstract

**Background:**

Conducting a conversation about wishes for treatment at end of life (EOL) has been shown to improve EOL care for patients and relatives. Still, only a minority of physicians conduct the conversation, which might lead to unwanted interventions and treatments.

**Objectives:**

The purpose of this survey was to examine which factors facilitate and hinder physicians across a wide range of health care facilities to initiate the conversation about wishes for treatment at EOL.

**Design:**

A questionnaire survey based on a Delphi-developed questionnaire.

**Setting/Subjects:**

The questionnaire was sent to both hospital physicians and general practitioners (GPs) in a Danish region.

**Results:**

More than 3000 physicians were invited to participate in the survey. Of these, 782 responded, 622 working at a hospital department, and 160 from general practice clinics. Results showed that senior physicians, GPs, and physicians working in a medical department feel best equipped to conduct the conversation. Moreover, senior physicians pointed to their experience as physicians as being of great importance for conducting the conversation, whereas junior physicians found training in conducting the conversation as an important factor.

**Conclusion:**

Our study indicates that different factors depending on the health care setting and the seniority of the physician facilitate or hinder physicians from conducting the conversation about wishes for treatment at EOL. Being aware of these differences and making a concerted effort depending on setting and seniority might help implement and conduct the conversation.

## Introduction

The wish to have some influence and control over their treatment at end of life (EOL) is increasing among patients,^1^ and one of the methods to secure this is through advance care planning (ACP). ACP is the process of talking about and documenting the prognosis, priorities, and preferences for goals of care at EOL as well as the hopes and fears regarding death and dying.^1–3^ Guidelines recommend conducting ACP with terminal patients with a life expectancy of less than a year.^2,4^ Most evidence demonstrates that early ACP and discussions of EOL care with patients with a life-threatening illness and their families have several beneficial factors for both patients and relatives.^5^

Early studies showed that the conversation about wishes for treatment at EOL increases quality of life (QoL) near death, causes less distress among caregivers, and leads to better compliance with the patient's wishes, thereby reducing the risk of unwanted interventions and treatments.^2,6^ Later studies showed less distress and better satisfaction among patients and relatives who engaged in EOL conversations but did not find the same results regarding increased QoL.^7,8^

With the growing and aging population worldwide, the demand for ACP and EOL discussions cannot be met by specialists of palliative care (PC) alone but also falls to generalists.^1,9^ It is therefore essential that both palliative specialists and generalists in different health care settings feel capable of conducting an EOL conversation and are attentive to when to initiate the conversation. However, despite the abovementioned positive findings, only a minority of physicians perform these conversations in practice.^10,11^ A recent review found that education level and time spent working with dying patients enhanced PC knowledge and self-efficacy. The perception of self-efficacy was linked to the physicians' sense of feeling able and prepared to handle EOL conversations, and was improved after the observation of senior colleagues.^12^

Some of the barriers for conducting the conversation reported in studies include prognostic uncertainty, navigating patient readiness, fear of the impact on patients and caretakers, feeling unaccustomed to such discussions, and lack of training and education.^1,13^ In 2014, a study identified 90 different barriers related to the knowledge, attitudes, and practices of intensive care unit physicians when providing EOL care concerning communication and decision making.^14^

In 2017, a study investigating the attitudes to and barriers for PC and ACP among pulmonologists found lack of time and staff and unpredictability of prognosis as the main barriers, but in general the attitude toward ACP and PC was positive and implementation was regarded as important.^10^ In acute care settings, the barriers related to EOL care and conversations were found to be the disagreement between physicians about a patient's transition from curative care to PC and families, or patients having unrealistic expectations for a treatment or not understanding the terminal state of a disease.^15^

Other studies have emphasized general practitioners (GPs) as having a leading role in encouraging and engaging ACP and EOL conversations because of their longstanding patient–GP relationship and knowledge of the patient.^16^ But barriers such as lack of skills to deal with patient's request and difficulties ensuring the wishes are respected also make it difficult for GPs to conduct the EOL conversation.^16,17^

The abovementioned studies suggest that there might be a difference in the facilitators and barriers depending on settings and physicians' experiences. This indicates a need for further investigation on the subject to be able to make a concerted effort to implement ACP conversations in a variety of settings.

The purpose of this survey was to examine which factors facilitate and hinder physicians across a wide range of departments and both primary and secondary care facilities to initiate the conversation about wishes for treatment at EOL. It also aimed to examine the association between the identified factors and demographic variables.

## Method

### Design

Questionnaire survey.

### Development of the questionnaire

The questionnaire was developed through a Delphi method.^18–20^ The Delphi panel consisted of nine physicians from both general practice and hospitals, including medical and surgical departments. Three of the panelists were women. The composition of the group included experienced physicians who have daily conversations with patients and relatives about EOL and ACP as well as younger physicians with less experience in EOL discussions and ACP but with an interest or scientific background in EOL discussions. The Delphi processing group consisted of the author group.

**FIG. 1.**
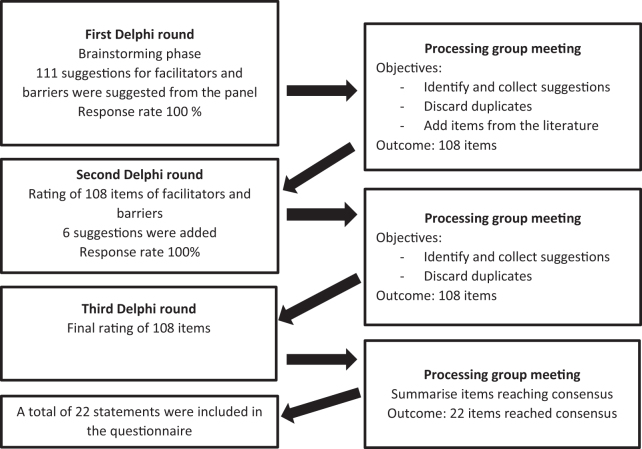
Flowchart of the Delphi process.

The Delphi process consisted of three rounds (Fig. 1) starting with two open-ended questions: “(1) What do you think facilitates physicians to conduct the conversation about wishes for treatment at end-of-life? (2) What do you think hinders physicians from conducting the conversation about wishes for treatment at end-of-life?” After the first round, duplicates were removed, and different understandings of the suggestions were discussed and resolved in the processing group. Furthermore, a list of items found in the literature (14) was discussed and added to the suggested items for the second Delphi round, thus synthesizing a list of 108 proposed facilitating and hindering items (Fig. 1).

In round two, the Delphi panel was asked to rate each item for relevance in conducting the conversation about wishes for treatment at EOL on a five-point Likert scale: 1 = *not relevant*, 2 = *less relevant*, 3 = *relevant*, 4 = *very relevant,* and 5 = *essential*. The panel participants also had the opportunity to comment and suggest new items if they found some were missing. In the processing of round two, the new item submissions were discussed in the processing group following the same procedure as in round one. Six new suggestions were added after round two, but all were duplicates and discarded.

In round three, the Delphi panel was presented with the new items and their prior ratings of the other factors and asked to rate the items once more. In this round, it was not possible for the panelists to comment or add new suggestions. After round three, the processing group met and summarized the items that reached a consensus.

A predefined consensus rank was used to find the relevant items, and a consensus was obtained when >60% of the participants in the Delphi panel had rated the item as 4 = *very relevant* or 5 = *essential*. All items that did not reach a consensus were subsequently excluded. The suggestions and ratings of each participant were known only by the first author, and were anonymous to the processing group and the Delphi panel throughout the study.

The statements in the questionnaire were formulated based on the 22 items that reached consensus in the Delphi process. The items were transformed into statements by the first author, and then discussed and adjusted by the process group. Furthermore, some contextual questions such as gender, workplace, and seniority were added to the first part of the questionnaire, and the respondents had the opportunity to write comments at the end of the questionnaire. After the process group approved the content of the contextual questions and formulation of the statements, the questionnaire was pilot-tested for face and content validity with the help of seven physicians. The testing did not result in any changes (Supplementary Data S1).

### Respondents

As we wanted to include as wide a sample as possible, we invited all clinical departments and general practice clinics (GPCs) in the region to participate in the survey. No purposive sampling was applied before approaching the hospital departments.

The region, where the survey was conducted, is one of five regions in Denmark, and the population is considered representative of the background population. In Denmark, the GPs treat patients of all ages and a variety of diseases, whereas the hospital departments only examine selected group of patients related to their specialties.

The questionnaire was sent by e-mail between November 1, 2021 and January 24, 2022 to physicians working in a clinical department whose head of department had agreed to participate in the survey before the distribution of the questionnaire. The included departments were all from hospitals in the same region of Denmark. Each recipient either received a personal link to the survey, or their department received a public link to distribute to their physicians according to departmental wishes. If the recipients who received a personal link had not responded within 2 weeks, a reminder was sent. Recipients were registered as nonrepliers if they had not answered within 2 weeks after the last reminder.

All GPCs in the region with ∼1200 physicians received a letter by regular mail with information about the project, one to four paper questionnaires depending on the number of physicians in the clinic, a return envelope and a link to the survey.

In Denmark, all physicians have a communication course in their first year after medical school. The curriculum in PC and communication differs from the four medical schools in Denmark and which extra PC training or communication training physicians have received after medical school depends on specialty and the physician's own interest in the subject.

### Data collection and analyses

All data were collected, managed, and analyzed using the RedCap electronic data capture tool hosted by the Open Patient data Explorative Network (OPEN) and analyzed using the statistical program BE Stata 17.0. No sample size analysis was conducted, as all physicians from clinical departments and GPCs in the region were invited to participate. Results are presented using descriptive statistics with *n* (%). The chi-square test was used for group comparison (hospital physicians vs. GPs, junior vs. senior physicians, and physicians working in medical vs. surgical departments).

For comparative analyses, hospital physicians were assembled in either “Medical departments” or “Surgical departments.” Some respondents reported working in a hospital but in a different department than those listed in the questionnaire. Of these, most replied working within a medical specialty (rheumatology), and therefore all “others” were included in “Medical departments.”

A *p*-value <0.05 was considered statistically significant.

## Ethics

According to Danish legislation, this study did not need permission from The Regional Committees on Health Research Ethics. To ensure data security, a license agreement was obtained with OPEN (OP1202). The project was registered with the Danish Data Protection Agency (file number 20/44800). Each physician agreed to participate by completing the questionnaire.

## Results

Approximately 1900 physicians from four different hospitals (both urban and university hospitals) and 352 GPCs with ∼1200 physicians from the same region in Denmark were invited to participate in the survey. From the hospitals, 1778 physicians received a personal link to the survey, whereas three departments with estimated 30–50 physicians at each department received a public link for distribution to their physicians. The questionnaire was returned by 783 physicians. One respondent had not answered any questions and was excluded from the analyses. Of the 782 remaining physicians, 617 (79%) worked in a hospital, 160 (20%) as a GP, and 5 (1%) as “Other.” The total response rate was ∼25%. For hospital physicians the response rate was ∼32% (not knowing exactly how many physician received and answered a public link from their department), and for GPs it was ∼13%.

The five respondents answering “Other” as workplace were added to “Hospitals” in Table 1, as most of them had registered as working at a specific hospital department.

**Table 1.** Characteristics of the Respondents

Variables, ***n*** (%)^a^	All	GP	Hospitals
Respondents^b^	782	160 (20)	622 (79)
Female	432 (56)	101 (64)	327 (54)
Seniority (years)			
<3	98 (12)	8 (5)	90 (15)
>3 ≤ 6	89 (11)	4 (3)	84 (14)
>6 ≤ 10	81 (10)	14 (9)	64 (10)
>10 ≤ 20	240 (31)	71 (44)	169 (27)
>20	274 (35)	63 (39)	210 (34)
Senior physicians^c^	546 (70)	145 (91)	399 (65)
Numbers of respondents from medical departments			
Emergency medicine			26 (6)
Anesthesiology and intensive care			123 (28)
Endocrinology			18 (4)
Gastroenterology and hepatology			14 (3)
Geriatric			25 (6)
Hematology			20 (4)
Infectious diseases			14 (3)
Cardiology			41 (9)
Pulmonology			25 (6)
Nephrology			13 (3)
Neurology			21 (5)
Oncology			43 (10)
Pediatric			12 (3)
Internal medicine			31 (7)
Others			20 (4)
Total			446
Numbers of respondents from surgical department			
Gynecology and obstetrics			37 (22)
Vascular surgery			14 (8)
Neurosurgery			1 (0)
Ophthalmology			1 (0)
Gastrointestinal surgery			15 (9)
Orthopedics			54 (32)
Oto-rhino-laryngology			26 (15)
Plastic surgery			5 (3)
Thoracic surgery			9 (5)
Urology			9 (5)
Total			171
Have you received teaching in conducting the conversation about EOL? (Possible to choose more than one answer)			
Yes, by traditional apprenticeship	340 (29)	64 (40)	273 (44)
Yes, by theoretical presentations	354 (30)	99 (62)	253 (41)
Yes, by e-learning or similar	35 (3)	8 (5)	27 (4)
Yes, as self-study	153 (13)	34 (21)	118 (19)
No, has received no teaching	259 (22)	32 (20)	226 (37)
Other	41 (3)	13 (8)	27 (4)

^a^ Different *n* due to missing data.

^b^ Five respondents responded “Others” as workplace.

^c^ Senior physicians: physicians who have finished their specialization.

EOL, end of life; GP, general practitioners.

Table 1 shows the characteristics of the respondents, including all the specialties represented and the number of respondents from each specialty. Gender was equally distributed overall but with an overweight of female GPs. The majority of participants (66%) had >10 years of experience as physicians, which corresponds to the majority, especially for GPs (73%), being senior physicians (had finished their specialization). More hospital physicians than GPs had not received any training in conducting a conversation about wishes for treatment at EOL, and very few used an ACP document when conducting the conversation.

Table 2 shows comparisons between hospital physicians versus GPs, junior versus senior physicians, and physicians working in medical versus surgical departments when asked about the items “I feel well prepared to conduct the conversation about EOL with the patient and optionally a relative” and “I always or usually conduct the conversation when it is relevant.” GPs, senior physicians, and physicians from medical departments were significantly more likely to answer “Agree” or “Strongly agree” for both statements.

**Table 2.** Comparison on “Feeling Equipped to Conduct the Conversation” and “Always or Almost Always Conduct the Conversation” between the Different Groups of Physicians

Questions	Answers							
	Strongly disagree, ***n*** (%)	Disagree, ***n*** (%)	Neither agree or disagree, ***n*** (%)	Agree, ***n*** (%)	Strongly agree, ***n*** (%)	Unsure/don't know, ***n*** (%)	Total, ***n***^a^	***p*** ^b^
I feel well equipped to conduct the conversation								
General practitioners	1 (1)	7 (4)	20 (13)	70 (44)	60 (38)	0	158	0.002
Hospital physicians	15 (2)	72 (12)	115 (19)	223 (36)	177 (29)	10 (2)	612	
Senior physicians	6 (1)	23 (4)	75 (14)	212 (39)	214 (40)	8 (1)	538	<0.001
Junior physicians	10 (4)	56 (24)	60 (26)	81 (35)	23 (10)	2 (1)	232	
Medical department	8 (2)	44 (10)	78 (18)	164 (37)	139 (32)	7 (2)	440	0.033
Surgical department	7 (4)	27 (16)	37 (22)	56 (34)	37 (22)	3 (2)	167	
I always conduct the conversation when relevant								
General practitioners	0	2 (1)	21 (13)	69 (43)	66 (42)	1 (0)	159	0.005
Hospital physicians	11 (2)	32 (5)	84 (14)	259 (42)	195 (32)	31 (5)	612	
Senior physicians	8 (1)	12 (2)	57 (11)	220 (41)	217 (40)	23 (4)	537	<0.001
Junior physicians	3 (1)	22 (9)	48 (21)	107 (46)	44 (19)	9 (4)	233	
Medical department	5 (1)	24 (5)	53 (12)	193 (44)	152 (34)	14 (3)	441	0.001
Surgical department	6 (4)	8 (5)	31 (19)	65 (39)	41 (25)	15 (9)	166	

^a^ Different *n* due to missing data.

^b^ Based on chi-square test.

Physicians in hospitals, senior physicians, and physicians from medical departments were more likely to conduct the conversation about treatment at EOL compared with GPs, junior physicians, and surgical departments, respectively (Table 3).

**Table 3.** Comparison of How Often General Practitioners, Hospital, Senior Physicians, Junior Physicians, and Medical/Surgical Departments Conduct the Conversation About Wishes for Treatment in End of Life

How often do you conduct the conversation about wishes for EOL?	Daily, ***n*** (%)	Weekly, ***n*** (%)	Monthly, ***n*** (%)	3–6 times a year, ***n*** (%)	<3 times a year, ***n*** (%)	Never, ***n*** (%)	Total, ***n***^a^	***p*** ^b^
Health care facility								
General practitioners	6 (4)	23 (15)	74 (47)	45 (28)	9 (6)	1 (1)	158	<0.001
Hospital physicians	63 (10)	141 (23)	178 (29)	88 (14)	86 (14)	63 (10)	619	
Seniority								
Senior physicians	56 (10)	108 (20)	176 (33)	100 (18)	54 (10)	47 (9)	541	0.010
Junior physicians	13 (6)	55 (23)	76 (32)	33 (14)	41 (17)	17 (7)	235	
Departments								
Medical departments	46 (10)	128 (29)	135 (30)	63 (14)	42 (9)	29 (7)	443	<0.001
Surgical departments	17 (10)	12 (7)	43 (25)	25 (15)	41 (24)	33 (19)	171	

^a^ Different *n* due to missing data.

^b^ Chi-square test.

Of the 22 statements in the questionnaire (referred to as S1–S22), 12 statements showed no significant difference between the different groups of physicians who agreed that the statements were important factors in conducting the conversation (Table 4).

**Table 4.** Comparison of the 22 Statements in the Questionnaire between the Groups of Physicians: General Practitioners, Hospital Physicians, Senior Physicians, Junior Physicians, Physicians Working in Medical Departments, and Physicians Working in Surgical Departments

Questions	Answers							
	Strongly disagree, ***n*** (%)	Disagree, ***n*** (%)	Neither agree or disagree, ***n*** (%)	Agree, ***n*** (%)	Strongly agree, ***n*** (%)	Unsure/don't know, ***n*** (%)	Total, ***n***^a^	***p*** ^b^
1. I feel capable because of my own life experience								
General practitioners	3 (2)	8 (5)	23 (15)	79 (50)	45 (28)	0	158	0.015
Hospital physicians	16 (3)	65 (11)	115 (20)	235 (40)	141 (24)	13 (2)	585	
Senior physicians	6 (1)	25 (5)	80 (15)	237 (46)	164 (32)	6 (1)	518	<0.001
Junior physicians	13 (6)	48 (21)	58 (26)	76 (34)	22 (10)	7 (3)	224	
Medical departments	9 (2)	44 (10)	84 (20)	169 (40)	109 (26)	8 (2)	423	0.361
Surgical departments	7 (4)	20 (13)	30 (19)	65 (41)	31 (20)	5 (3)	158	
2. Experience with similar conversations								
General practitioners	2 (1)	5 (3)	14 (9)	79 (50)	57 (36)	0	157	0.029
Hospital physicians	13 (2)	56 (10)	72 (12)	262 (45)	172 (30)	7 (1)	582	
Senior physicians	4 (1)	16 (3)	44 (9)	247 (48)	199 (39)	5 (1)	515	<0.001
Junior physicians	11 (5)	45 (20)	42 (19)	94 (42)	29 (13)	2 (1)	223	
Medical departments	6 (1)	30 (7)	47 (11)	199 (47)	134 (32)	4 (1)	420	0.001
Surgical departments	7 (4)	25 (16)	25 (16)	61 (39)	37 (23)	3 (2)	158	
3. Knowing the patient's possibilities/opportunities								
General practitioners	1 (1)	0	3 (2)	60 (38)	92 (58)	2 (1)	158	0.864
Hospital physicians	2 (0)	3 (1)	17 (3)	220 (38)	329 (57)	11 (2)	582	
Senior physicians	3 (1)	2 (0)	16 (3)	198 (38)	285 (55)	11 (2)	515	0.480
Junior physicians	0	1 (0)	4 (2)	82 (37)	135 (60)	2 (1)	224	
Medical departments	1 (0)	1 (0)	12 (3)	163 (39)	241 (57)	4 (1)	422	0.055
Surgical departments	1 (0)	2 (1)	5 (3)	56 (36)	85 (54)	7 (4)	156	
4. Knowing how the patient's EOL will be								
General practitioners	0	0	15 (10)	57 (36)	81 (52)	4 (3)	157	0.184
Hospital physicians	2 (0)	4 (1)	40 (7)	268 (46)	259 (45)	9 (2)	582	
Senior physicians	2 (0)	3 (1)	37 (7)	229 (44)	233 (45)	11 (2)	515	0.748
Junior physicians	0	1 (0)	18 (8)	96 (43)	106 (48)	2 (1)	223	
Medical departments	2 (0)	2 (0)	29 (7)	195 (46)	188 (45)	5 (1)	421	0.659
Surgical departments	0	2 (1)	11 (7)	73 (47)	67 (43)	4 (3)	157	
5. My clinical experience helps me								
General practitioners	1 (1)	0	7 (4)	75 (47)	75 (47)	0	158	0.026
Hospital physicians	0	16 (3)	39 (7)	268 (46)	249 (42)	11 (2)	583	
Senior physicians	1 (0)	8 (2)	22 (4)	227 (44)	252 (49)	7 (1)	517	<0.001
Junior physicians	0	8 (4)	24 (11)	116 (52)	71 (32)	4 (2)	223	
Medical departments	0	7 (2)	22 (5)	204 (48)	182 (43)	6 (1)	421	0.003
Surgical departments	0	9 (6)	17 (11)	63 (40)	64 (41)	5 (3)	158	
6. Feeling it would be beneficial for the patient								
General practitioners	0	0	4 (3)	61 (39)	91 (58)	2 (1)	158	0.082
Hospital physicians	1 (0)	6 (1)	43 (8)	238 (42)	276 (48)	13 (2)	573	
Senior physicians	1 (0)	5 (1)	29 (6)	199 (39)	263 (52)	13 (3)	510	0.253
Junior physicians	0	1 (0)	18 (8)	100 (45)	103 (46)	2 (1)	224	
Medical departments	1 (0)	4 (1)	32 (8)	171 (41)	205 (49)	5 (1)	418	0.097
Surgical departments	0	2 (1)	11 (7)	67 (43)	67 (43)	8 (5)	155	
7. Believes sick/old/dying patients should have the opportunity to make their own decisions								
General practitioners	0	2 (1)	2 (1)	49 (31)	102 (65)	3 (2)	158	0.460
Hospital physicians	1 (0)	4 (1)	23 (4)	184 (32)	349 (60)	19 (3)	580	
Senior physicians	1 (0)	2 (0)	14 (3)	162 (32)	321 (62)	14 (3)	514	0.194
Junior physicians	0	4 (2)	11 (5)	71 (32)	129 (58)	8 (4)	223	
Medical departments	1 (0)	3 (1)	18 (4)	132 (31)	254 (60)	12 (3)	420	0.870
Surgical departments	0	1 (1)	5 (3)	52 (33)	91 (58)	7 (4)	156	
8. I feel comfortable conducting the conversation								
General practitioners	0	3 (2)	10 (6)	54 (34)	89 (56)	2 (1)	158	0.064
Hospital physicians	2 (0)	6 (1)	65 (11)	238 (41)	254 (44)	13 (2)	578	
Senior physicians	1 (0)	3 (1)	47 (9)	200 (39)	252 (49)	11 (2)	514	0.059
Junior physicians	1 (0)	6 (3)	28 (13)	92 (42)	90 (41)	4 (2)	221	
Medical departments	0	5 (1)	44 (11)	174 (42)	187 (45)	6 (1)	416	0.043
Surgical departments	2 (1)	1 (1)	21 (13)	62 (39)	65 (41)	7 (4)	158	
9. Teaching or training would help me/has helped me								
General practitioners	0	7 (4)	29 (18)	71 (45)	46 (29)	5 (3)	158	0.051
Hospital physicians	8 (1)	27 (5)	123 (21)	190 (32)	199 (34)	36 (6)	583	
Senior physicians	6 (1)	28 (5)	126 (24)	193 (37)	134 (26)	30 (6)	517	<0.001
Junior physicians	2 (1)	6 (3)	26 (12)	67 (30)	111 (50)	11 (5)	223	
Medical departments	4 (1)	19 (5)	90 (21)	135 (32)	153 (36)	20 (5)	421	0.126
Surgical departments	4 (3)	8 (5)	33 (21)	53 (34)	45 (28)	15 (9)	158	
10. Interdisciplinary involvement								
General practitioners	1 (1)	13 (8)	29 (18)	53 (34)	61 (39)	1 (0)	158	<0.001
Hospital physicians	4 (1)	12 (2)	67 (12)	178 (31)	306 (53)	13 (2)	580	
Senior physicians	3 (1)	22 (4)	69 (13)	178 (35)	233 (45)	10 (2)	515	0.005
Junior physicians	2 (1)	3 (1)	27 (12)	53 (24)	133 (60)	4 (2)	222	
Medical departments	3 (1)	9 (2)	48 (11)	125 (30)	228 (54)	6 (1)	419	0.266
Surgical departments	1 (1)	3 (2)	19 (12)	53 (34)	74 (47)	7 (4)	157	
11. Knowing the patient								
General practitioners	0	0	4 (3)	38 (24)	117 (74)	0	159	<0.001
Hospital physicians	3 (1)	24 (4)	102 (18)	186 (32)	257 (44)	9 (2)	581	
Senior physicians	1 (0)	16 (3)	76 (15)	146 (28)	270 (52)	7 (1)	516	0.315
Junior physicians	2 (1)	8 (4)	30 (13)	78 (35)	103 (46)	2 (1)	223	
Medical departments	2 (0)	18 (4)	73 (17)	129 (31)	193 (46)	4 (1)	419	0.351
Surgical departments	1 (1)	6 (4)	29 (18)	55 (35)	62 (39)	5 (3)	158	
12. Having time for the conversation								
General practitioners	0	1 (1)	1 (1)	28 (18)	127 (80)	1 (1)	158	0.600
Hospital physicians	1 (0)	3 (1)	15 (3)	111 (19)	443 (76)	9 (2)	582	
Senior physicians	1 (0)	4 (1)	12 (2)	105 (20)	387 (75)	6 (1)	515	0.332
Junior physicians	0	0	4 (2)	34 (15)	182 (81)	4 (2)	224	
Medical departments	1 (0)	2 (0)	11 (3)	83 (20)	317 (75)	6 (1)	420	0.972
Surgical departments	0	1 (1)	4 (3)	28 (18)	122 (77)	3 (2)	158	
13. Possibility to conduct the conversation in privacy or in a conversation room								
General practitioners	1 (1)	1 (1)	14 (9)	28 (18)	99 (63)	3 (2)	158	0.531
Hospital physicians	3 (0)	5 (1)	37 (6)	129 (22)	396 (69)	9 (2)	578	
Senior physicians	3 (1)	4 (1)	34 (7)	121 (24)	343 (67)	8 (2)	513	0.846
Junior physicians	0	1 (0)	17 (8)	49 (22)	151 (68)	4 (2)	222	
Medical departments	2 (0)	4 (1)	25 (6)	88 (21)	293 (70)	6 (1)	418	0.596
Surgical departments	0	1 (1)	12 (8)	41 (26)	99 (63)	3 (2)	156	
14. Indicating to have time (e.g., by sitting down)								
General practitioners	0	0	3 (2)	37 (23)	116 (73)	3 (2)	159	0.703
Hospital physicians	1 (0)	5 (1)	20 (3)	143 (25)	402 (69)	10 (2)	581	
Senior physicians	1 (0)	1 (0)	14 (3)	132 (26)	358 (70)	9 (2)	515	0.140
Junior physicians	0	4 (2)	9 (4)	48 (21)	159 (71)	4 (2)	224	
Medical departments	1 (0)	3 (1)	12 (3)	103 (24)	296 (70)	6 (1)	421	0.576
Surgical departments	0	2 (1)	8 (5)	40 (26)	102 (65)	4 (3)	156	
15. When resources are allocated								
General practitioners	0	5 (3)	8 (5)	43 (27)	99 (63)	3 (2)	158	0.272
Hospital physicians	2 (0)	6 (1)	49 (8)	155 (27)	355 (61)	14 (2)	581	
Senior physicians	1 (0)	7 (1)	42 (8)	139 (27)	313 (61)	12 (2)	514	0.955
Junior physicians	1 (0)	4 (2)	15 (7)	59 (26)	140 (63)	5 (2)	224	
Medical departments	2 (0)	4 (1)	39 (9)	111 (26)	256 (61)	8 (2)	420	0.571
Surgical departments	0	2 (1)	10 (6)	44 (28)	95 (61)	6 (4)	157	
16. When the patient initiates the conversation								
General practitioners	0	5 (3)	33 (21)	59 (37)	61 (38)	1 (1)	159	0.181
Hospital physicians	5 (1)	6 (1)	110 (19)	216 (37)	230 (40)	15 (3)	582	
Senior physicians	3 (1)	8 (2)	106 (21)	198 (38)	188 (36)	13 (3)	516	0.218
Junior physicians	2 (1)	3 (1)	37 (17)	76 (34)	103 (46)	3 (1)	224	
Medical departments	3 (1)	5 (1)	89 (21)	147 (35)	169 (40)	8 (2)	421	
Surgical departments	2 (1)	1 (1)	21 (13)	68 (43)	58 (37)	7 (4)	157	0.084
17. Easier conducting the conversation with older patients								
General practitioners	1 (1)	6 (4)	29 (18)	45 (28)	69 (43)	9 (6)	159	0.275
Hospital physicians	3 (1)	29 (5)	146 (25)	175 (30)	196 (34)	34 (6)	583	
Senior physicians	3 (1)	29 (6)	133 (26)	157 (30)	165 (32)	30 (6)	517	0.019
Junior physicians	1 (0)	6 (3)	42 (19)	62 (28)	100 (45)	13 (6)	224	
Medical departments	3 (1)	24 (6)	113 (27)	115 (27)	149 (35)	18 (4)	422	0.009
Surgical departments	0	5 (3)	33 (21)	57 (36)	46 (29)	16 (10)	157	
18. The patient has an understanding of the severity of the disease								
General practitioners	0	1 (1)	5 (3)	48 (30)	104 (65)	1 (1)	159	0.352
Hospital physicians	0	4 (1)	20 (3)	203 (35)	338 (58)	16 (3)	581	
Senior physicians	0	2 (0)	21 (4)	181 (35)	299 (58)	12 (2)	515	0.202
Junior physicians	0	3 (1)	4 (2)	70 (31)	142 (63)	5 (2)	224	
Medical departments	0	3 (1)	16 (4)	137 (33)	258 (61)	7 (2)	421	0.015
Surgical departments	0	1 (1)	4 (3)	64 (41)	78 (50)	9 (6)	156	
19. Good chemistry between me (the physician) and the patient								
General practitioners	0	2 (1)	10 (6)	50 (32)	95 (60)	1 (1)	158	<0.001
Hospital physicians	2 (0)	16 (3)	78 (14)	259 (45)	212 (37)	13 (2)	580	
Senior physicians	0 (0)	12 (2)	56 (11)	218 (42)	218 (42)	10 (2)	514	0.245
Junior physicians	2 (1)	6 (3)	32 (14)	91 (41)	88 (38)	4 (2)	223	
Medical departments	1 (0)	11 (3)	56 (13)	189 (45)	155 (37)	7 (2)	419	0.665
Surgical departments	1 (1)	5 (3)	22 (14)	68 (43)	55 (35)	6 (4)	157	
20. When the relatives are present								
General practitioners	1 (1)	2 (1)	43 (27)	64 (41)	46 (29)	1 (1)	157	0.527
Hospital physicians	2 (0)	8 (1)	144 (25)	225 (39)	185 (32)	19 (3)	583	
Senior physicians	2 (0)	9 (2)	129 (25)	203 (39)	159 (31)	13 (3)	515	0.794
Junior physicians	1 (0)	1 (0)	58 (26)	85 (38)	72 (32)	7 (3)	224	
Medical departments	2 (0)	6 (1)	98 (23)	158 (37)	148 (35)	10 (2)	422	0.037
Surgical departments	0	2 (1)	45 (29)	65 (41)	36 (23)	9 (6)	157	
21. Difficulties assessing when the conversation is relevant/should be conducted								
General practitioners	16 (10)	65 (41)	42 (26)	30 (19)	5 (3)	1 (1)	159	0.713
Hospital physicians	52 (9)	232 (40)	150 (26)	109 (19)	19 (3)	17 (3)	580	
Senior physicians	59 (11)	236 (46)	135 (26)	59 (11)	13 (3)	13 (3)	515	<0.001
Junior physicians	9 (4)	61 (27)	57 (26)	80 (36)	11 (5)	5 (2)	223	
Medical departments	44 (11)	181 (43)	101 (24)	71 (17)	14 (3)	7 (2)	418	0.001
Surgical departments	8 (5)	51 (32)	47 (30)	37 (23)	5 (3)	10 (6)	158	
22. No time or resources to conduct the conversation								
General practitioners	22 (14)	66 (42)	38 (24)	24 (15)	9 (6)	0	159	0.004
Hospital physicians	54 (9)	178 (31)	180 (31)	94 (16)	52 (9)	24 (4)	582	
Senior physicians	66 (13)	193 (37)	142 (22)	71 (14)	30 (6)	15 (3)	517	<0.001
Junior physicians	10 (4)	50 (22)	76 (34)	47 (21)	31 (14)	9 (4)	223	
Medical departments	45 (11)	137 (32)	124 (29)	70 (17)	32 (8)	14 (3)	422	0.078
Surgical departments	9 (6)	41 (26)	54 (35)	24 (15)	19 (12)	9 (6)	156	

The questionnaire in full text can be seen in Supplementary Data S1.

^a^ Different *n* due to missing data.

^b^ Chi-square test.

There was a difference between senior and junior physicians when asked about the importance of conducting the conversation for wishes at EOL depending on “Their own life experience” (S1) and “Experience with similar conversations” (S2). In both cases, significantly more senior physicians agreed that both statements (S1 and S2) helped them conduct the conversation compared with junior physicians.

Similarly, more senior physicians agreed their “Clinical experience helps me” (S5) conduct the conversation. For these statements, no statistical difference was found when comparing GPs and hospital physicians or medical and surgical departments. Significantly, more junior physicians found that training would help them conduct the conversation. Likewise, junior physicians to a higher extent agreed that “Interdisciplinary involvement” (S10) and “Easier to conduct the conversation with older patients” (S17) helped them conduct the conversation compared with senior physicians.

When comparing GPCs and hospitals, the main differences were found for “Interdisciplinary involvement” (S10), “Knowing the patient” (S11), and having “Good chemistry between the physician and patient” (S19), showing that GPs agreed “Knowing the patient” and “Having good chemistry” as more important than hospital physicians. “Interdisciplinary involvement” was more important to hospital physicians than GPs.

When comparing medical and surgical departments, statistical differences were found in the statements regarding “Experience with similar conversations” (S2) and “Clinical experience” (S5), both of which seemed to be more helpful for physicians in medical departments when conducting the conversation. By contrast, physicians in surgical departments to a higher extent agreed to have “Difficulties assessing when to conduct the conversation” (S21).

The majority of physicians did not use an ACP document during the conversations about wishes for EOL. Those using an ACP document were mostly senior physicians and physicians at medical departments (Table 5).

**Table 5.** Comparison of Using an Advance Care Planning Document during End-of-Life Conversations

During the conversation, do you use an ACP document	Yes	No
Total, *n* (%)^a^	36 (5)	676 (95)
Health care professionals, *n* (%)		
General practitioners	11 (7)	145 (93)
Hospital physicians	25 (5)	527 (95)
Seniority, *n* (%)		
Senior physicians	32 (6)	461 (94)
Junior physicians	4 (2)	214 (98)
Departments, *n* (%)		
Medical departments	22 (5)	392 (95)
Surgical departments	3 (2)	135 (98)

^a^ Different *n* due to missing data.

ACP, advance care planning.

In total, 141 respondents added a comment, mostly regarding how they found the subject to be either important or not relevant for their specialty. One respondent (an anesthesiologist) wrote, “I conduct an enormous amount of these conversations. Thank you so much for the initiative. To me the biggest problem is that the conversation is first conducted when I arrive. At this point the patient is often acutely affected and has trouble participating. There ought to be an automatic process with patients with liver cirrhosis and COPD as well as patients >75 years old. These patients are often re-admitted and in an acute state where it is difficult to talk to them.”

Another commented, “I am an orthopaedic surgeon and never conduct the conversation about end of life and therefore all of the above questions are irrelevant to me.” A GP wrote in the commentary, “It is something where you have to TAKE the time in everyday life and other things must be de-prioritised.”

No new items of importance for conducting the conversation about wishes for EOL were described in the comments.

## Discussion

Our study indicates that different factors facilitate or hinder physicians in conducting the conversation about wishes for treatment at EOL depending on the health care setting and the seniority of the physician.

For physicians working in GPCs, the factors “Knowing the patient” and “Having a good chemistry between the physician and patient” were especially evaluated as factors of importance for conducting the conversation. Junior physicians strongly agreed that “Teaching in conducting the conversation” was of great importance, while senior physicians emphasized their “Clinical experience” and “Experience with similar conversations” as some of the most important factors.

In general, physicians working in GPCs felt better equipped to conduct the conversation about wishes for EOL, as did senior physicians compared with junior physicians. The results also indicate that physicians working in a medical department conduct the conversation about wishes for EOL more often compared with GPs and physicians working in surgical departments.

In approximately half of the statements, there were no significant differences between the groups (senior physicians, junior physicians, GPs and medical or surgical departments, and GPCs). This outcome was expected since the questionnaire was based on a consensus method. Thus, it confirms that the items found by the Delphi group are of importance for conducting the conversation about wishes for EOL across different health care settings and seniority.

In contrast to the results of a study by Sørensen et al,^10^ lack of time and resources did not seem to be a barrier in this study, where most physicians across groups disagreed that lack of time affected their ability to conduct the conversation about EOL. Our results do not explain this difference from the literature, but we speculate that the respondents might be a selected group of physicians who actually conduct the conversation, and therefore prioritize this in their workday and take the time necessary. Some of the comments from the respondents also substantiated this assumption.

Likewise, Sørensen et al. reported “Unpredictability of prognosis” as a main barrier to conduct an ACP conversation, whereas the results from this study showed that most respondents disagreed with having difficulties in assessing when to conduct the conversation. Only junior physicians deviated from this finding, which is expected considering their lack of clinical experience. This result is compatible with the findings from Sørensen et al. that younger physicians reported more barriers than experienced physicians did.^10^ Similarly—and not surprisingly—did junior physicians agree that teaching or training helped them conduct the conversation. Our results mirror former studies that found training in PC is sparse, and incorporating a formal curriculum into training programs is likely to be helpful.^21–23^

As seen in Table 5, the majority of physicians did not use an ACP document during the conversations about wishes for EOL. A study by Tuesen et al^24^ showed that most physicians found an ACP document useful for both structuring and executing the conversation. However, no formal ACP document is yet implemented nationwide in Denmark, and this might explain the lack of use.

This study showed a considerably lower percentage of surgeons conducting the conversation compared with the respondents from medical specialties, which corresponds well with the differences between medical and surgical departments when asked if they “Feel capable in conducting the conversation” and “Always conduct it when relevant.” This is in accordance with the study by Kalbfell et al,^25^ which showed that even though surgeons believe preoperative discussions of patients' preferences for postoperative life-sustaining treatments and ACP are important, only 6% of patients undergoing major surgery discussed these issues preoperatively with their surgeon.

It is seen in some of the comments from the respondents not all surgeons believe that EOL conversations are relevant for their specialty, which might also to some extent explain the lower percentages of surgeons conducting the conversation.

Our results indicate that there are differences in facilitators and barriers depending on specialty, setting, and seniority when physicians are conducting the conversation about EOL. For the junior physicians, this could be apprentice teaching in both GPCs and hospital settings, or as a competence area when qualifying for a residency. Also in the curriculum at medical school, it could be a mandatory area during communication classes. For the senior physician, it might more be a question of logistics and planning rather than more education in the area. Being aware of these differences and trying to make a targeted effort will be beneficial for implementing ACP conversations and helping physicians conduct conversations about EOL.

### Strengths and limitations

This survey has several strengths. The questionnaire was developed with a consensus method that included physicians with different experiences and from different specialties and settings. It was developed taking into account that it would be sent to different care facilities. The questionnaire was not too long, and could easily be completed either electronically or by paper. The department heads were informed beforehand and had the opportunity to inform the physicians about the upcoming survey.

Another strength of this survey is the diversity of included departments and respondents. The respondents came from many different medical and surgical specialties, included both junior and senior physicians, and represented both genders. This scope gives a unique insight into which specialties and health care settings the conversation about wishes and treatment at EOL is prioritized.

In this study, we did not define “The conversation about wishes for treatment in End of Life” more specifically, but left it to the participants to decide their interpretation of EOL conversations. This might give a wide and overlapping definition, and can be seen as both a strength and a limitation of the study. We wanted to get an impression of how much physicians engage in EOL conversations in different health settings. Therefore, in this study we see the wide definition of EOL conversation as a strength. It would be interesting in future research to investigate both when the different physicians feel the conversation is relevant, how they define the ACP/EOL conversation, and how they conduct the conversation. Qualitative studies are necessary to provide a more specific answer to these questions.

A substantial limitation of the study is the low response rate. This was partly expected since the questionnaire was sent to a wide range of physicians across many different specialties and facilities. It is reasonable to assume that the physicians who responded to the survey may have had a special interest in EOL conversations, and the nonrespondents were more likely to avoid ACP conversation or did not think about it as an important task in their specialty. Therefore, the results were likely subjected to selection bias and thus not fully representative.

Furthermore, it is interesting that more senior than junior physicians returned the questionnaire. Unfortunately, our data are not sufficient to investigate if the difference between senior and junior physicians is explained by the fact that there are proportionally more senior physicians than junior physicians, or because it is common practice in Denmark to have senior physicians decide any treatment limitations, and senior physicians thereby have felt more obligated to respond to the survey.

The analyses include multiple comparisons, which may induce type one errors.

Developing the questionnaire using a consensus method also has some limitations. The items listed are based on experts' opinions on the subject. With this method, there might be some items not included in the questionnaire that could have importance for novices but were left out by experts. Using a different composition of experts in the Delphi panel and applying some less experienced physicians would most likely have provided different items. Qualitative studies with both experienced and unexperienced physicians would be interesting in the future to see if there are factors which have not been emphasized in this study. However, although all participants, including the junior physicians, had the opportunity to write comments and point out lacking items, none did so.

## Conclusion

Most physicians across different health care facilities and seniority feel equipped to conduct a conversation about wishes for treatment in EOL and feel they conduct it when relevant. For senior physicians, their clinical experience and experience with similar conversations help them conduct the conversation, whereas junior physicians emphasize teaching or training in EOL conversations as beneficial factors. GPs highlight “Good chemistry between the patient and physician” and “Knowing the patient” as important factors, while “Interdisciplinary involvement” seems more important in hospital settings.

More studies need to be conducted to identify the facilitators and barriers in each specialty and health care setting to conduct a more focused effort in implementing EOL conversations and ACP.

## Supplementary Material

Supplemental data

## Abbreviations Used

ACP: advance care planning
EOL: end of life
GPs: general practitioners
GPCs: general practice clinics
ICU: intensive care unit
OPEN: Open Patient data Explorative Network
PC: palliative care
QoL: quality of life
