# Supplementary material for: Facilitators and Barriers for Initiating Conversations About End of Life
Source: Palliat Med Rep. 2022 Nov 21;3(1):296–307. doi: 10.1089/pmr.2022.0042 (PMC9805848; doi:10.1089/pmr.2022.0042)
Supplement: Supplemental data [file Suppl_DataS1.docx]

**Questionnaire about the conversation on wishes for treatment in End of Life (EOL)**

Dear colleague

The questionnaire consists of some general questions about you and a series of statements, where you are asked to indicate how important these are for you to conduct the conversation about wishes for treatment in end of life.

Thank you so much for taking the time to answer the survey.

Best regards

Mette Aaby Smith

Ph.D. student, MD

Department of Anesthesiology and Intensive Care Medicine, Kolding Hospital

Where are you employed?

□ General practice □ Hospital □ Other: _______________________________

In which specialty are you employed?

- Emergency medicine
- Anesthesiology and Intensive care
- Dermatology
- Endocrinology
- Gastroenterology and hepatology
- Geriatric
- Hematology
- Infectious Diseases
- Cardiology
- Pulmonology
- Nephrology
- Neurology
- Oncology
- Pediatric
- Internal medicine
- Gynecology and obstetrics
- Vascular surgery
- Neurosurgery
- Ophthalmology
- Gastrointestinal surgery
- Orthopedics
- Oto-rhino-laryngology
- Plastic surgery
- Thoracic surgery
- Urology
- Other: ________________________________________________

If answered “Hospital” under employment/question 1:

Where do you work most of your workinghours/time?

- On-call physician/prehospital physician
- Outpatient clinic physician
- Emergency room physician
- Intensive care physician
- In the surgical ward
- Ward physician
- Others: __________________________________________________

If answered “General practice” under employment Question 1:

Where do you work most of your workinghours/time?

- General practice
- On-call physician
- Other: _________________________________________________

Have you specialized (Senior physician) or are you a junior physician?

- Senior physician
- Junior physician

Any subspecialty: __________________________________________________

How many years of clinical experience do you have?

(number of years since graduating from medical school deducted years with research, maternity leave or similar)

- < 3 years
- > 3 ≤ 6 years
- > 6 ≤ 10 years
- > 10 ≤ 20 years
- > 20 years

What gender do you identify with?

- Female
- Male
- Other

How often do you conduct conversations about treatment in EOL with patients and relatives (if any)?

- Daily
- Weekly
- Monthly
- 3-6 times a year
- <3 times a year
- Never

Have you at some point received teaching in conducting the conversation about EOL/the necessary conversation? (without teaching in communication at medical school)

- Yes, by traditional apprenticeship
- Yes, by theoretical presentations
- Yes, by e-learning or similar
- Yes, as self-study
- No, has received no teaching
- Other: _________________________________________________

During the conversation, I use an Advance Care Planning document*, a conversation template or similar

- Yes
- No

*“Advance care planning/plan”: planning of future care and treatment and is a conversation between patient, health professionals and relatives, where the starting point is the patient’s wishes and thoughts for the end of life and death. The conversation is documented in the patient’s medical journal.

|  | Strongly disagree | Disagree | Neither disagree or agree | Agree | Strongly agree | Unsure/ Don’t know |
| --- | --- | --- | --- | --- | --- | --- |
| I feel well equipped/able to conduct the conversation about EOL with the patient and optionally a relative | ⃝ | ⃝ | ⃝ | ⃝ | ⃝ | ⃝ |
| I always or usually conduct the conversation when it is relevant | ⃝ | ⃝ | ⃝ | ⃝ | ⃝ | ⃝ |

|  | Strongly disagree | Disagree | Neither disagree or agree | Agree | Strongly agree | Unsure/ Don’t know |
| --- | --- | --- | --- | --- | --- | --- |
| 1. I feel capable to conduct the conversation about treatment in EOL because of my own life experience (based on private and professional experiences) | ⃝ | ⃝ | ⃝ | ⃝ | ⃝ | ⃝ |
| 2. Because of my experience with similar conversation, I feel well equipped to conduct the conversation about treatment in EOL | ⃝ | ⃝ | ⃝ | ⃝ | ⃝ | ⃝ |
| 3. It helps me conduct the conversation when I know which possibilities/opportunities the patient has in EOL | ⃝ | ⃝ | ⃝ | ⃝ | ⃝ | ⃝ |
| 4. It helps me, when I know how the patients EOL probably will be | ⃝ | ⃝ | ⃝ | ⃝ | ⃝ | ⃝ |
| 5. My clinical experience helps me conduct the conversation | ⃝ | ⃝ | ⃝ | ⃝ | ⃝ | ⃝ |
| 6. It helps me conduct the conversation when I have a feeling that it would be beneficial for the patient and possibly the relatives | ⃝ | ⃝ | ⃝ | ⃝ | ⃝ | ⃝ |
| 7. I believe that sick/old/dying patients should have the opportunity to make decisions about their own treatment in EOL, which helps me conduct the conversation about treatment in EOL | ⃝ | ⃝ | ⃝ | ⃝ | ⃝ | ⃝ |
| 8. It helps that I feel comfortable conducting the conversation about EOL | ⃝ | ⃝ | ⃝ | ⃝ | ⃝ | ⃝ |
| 9. Teaching or training in the conversation about EOL would help me/has helped me conducting the conversation | ⃝ | ⃝ | ⃝ | ⃝ | ⃝ | ⃝ |
| 10. It helps me conduct the conversation when it is possible for interdisciplinary involvement, e.g. by participation of a nurse or nursing staff | ⃝ | ⃝ | ⃝ | ⃝ | ⃝ | ⃝ |
| 11. It helps conduct the conversation, when I know the patient and the relatives (if any) | ⃝ | ⃝ | ⃝ | ⃝ | ⃝ | ⃝ |
| 12. It helps that I have time for the conversation | ⃝ | ⃝ | ⃝ | ⃝ | ⃝ | ⃝ |
| 13. It helps, that I have the possibility to conduct the conversation in privacy or a conversation room with the patient and the relatives (if any) | ⃝ | ⃝ | ⃝ | ⃝ | ⃝ | ⃝ |
| 14. It helps when I can indicate to have time for the conversation (for example by sitting down) | ⃝ | ⃝ | ⃝ | ⃝ | ⃝ | ⃝ |
| 15. It facilitates conducting the conversation when resources are allocated, for example extra personal, planning of time and place and so forth | ⃝ | ⃝ | ⃝ | ⃝ | ⃝ | ⃝ |
| 16. It helps when the patient initiates the conversation or expresses a wish for talking about EOL | ⃝ | ⃝ | ⃝ | ⃝ | ⃝ | ⃝ |
| 17. It is often easier to conduct the conversation about EOL with older patients | ⃝ | ⃝ | ⃝ | ⃝ | ⃝ | ⃝ |
| 18. It helps to conduct the conversation when the patient and the relatives (if any) have an understanding about the severity of the disease/condition | ⃝ | ⃝ | ⃝ | ⃝ | ⃝ | ⃝ |
| 19. It helps me conduct the conversation when there is a good chemistry between me (the physician) and the patient | ⃝ | ⃝ | ⃝ | ⃝ | ⃝ | ⃝ |
| 20. It helps me conduct the conversation about EOL when the relatives are present | ⃝ | ⃝ | ⃝ | ⃝ | ⃝ | ⃝ |
| 21. I find it difficult to assess when the conversation about EOL is relevant/should be conducted | ⃝ | ⃝ | ⃝ | ⃝ | ⃝ | ⃝ |
| 22. I do not feel there is the time or resources during ordinary working days to conduct the conversation | ⃝ | ⃝ | ⃝ | ⃝ | ⃝ | ⃝ |
|  |  |  |  |  |  |  |
